# Ketamine and α-Amino-3-Hydroxy-5-Methyl-4-Isoxazolepropionic Acid (AMPA) Receptor Potentiation in the Somatosensory Cortex: A Comprehensive Review

**DOI:** 10.7759/cureus.69261

**Published:** 2024-09-12

**Authors:** Kaustuv Das, Jayshree Sen, Aishwarya S Borode

**Affiliations:** 1 Anaesthesiology, Jawaharlal Nehru Medical College, Datta Meghe Institute of Higher Education and Research, Wardha, IND

**Keywords:** ampa receptors, ketamine, nmda receptor antagonism, sensory processing, somatosensory cortex, synaptic plasticity

## Abstract

Ketamine, a dissociative anesthetic primarily recognized for its antagonism of N-methyl-D-aspartate (NMDA) receptors, has gained significant attention for its rapid antidepressant effects and potential in treating mood disorders. However, recent research indicates that ketamine’s influence extends beyond NMDA receptor inhibition, affecting α-amino-3-hydroxy-5-methyl-4-isoxazolepropionic acid (AMPA) receptors and sensory processing. This review delves into ketamine's role in enhancing AMPA receptor function and its implications for sensory processing within the somatosensory cortex. AMPA receptors, essential for fast excitatory neurotransmission and synaptic plasticity, play a key role in sensory perception and integration. By examining preclinical and clinical studies, this review sheds light on how ketamine’s modulation of AMPA receptors may improve sensory processing and contribute to its therapeutic effects. Additionally, the review explores the potential for ketamine-based therapies to treat sensory processing disorders and refine current treatment strategies. A deeper understanding of ketamine’s complex effects on AMPA receptors and sensory processing could provide valuable insights for developing targeted interventions and advancing clinical applications.

## Introduction and background

Sensory processing is fundamental to how organisms perceive and respond to their environment. It involves the nervous system's detection, integration, and interpretation of sensory stimuli. In humans, sensory processing begins with activating sensory receptors that detect external stimuli such as touch, temperature, and pain [1]. These signals are transmitted to the central nervous system (CNS) through peripheral nerves, reaching various cortical regions for processing. Among these regions, the somatosensory cortex, located in the brain's parietal lobe, plays a crucial role. This area is organized into distinct regions corresponding to different body parts, forming a sensory map known as the somatotopic map. Neurons in the somatosensory cortex encode various aspects of sensory stimuli, such as intensity, location, and texture, which are essential for constructing a coherent sensory experience [2]. Alpha-amino-3-hydroxy-5-methyl-4-isoxazolepropionic acid (AMPA) receptors are ionotropic glutamate receptors integral to synaptic transmission and plasticity in the CNS. These receptors mediate fast excitatory synaptic transmission and are crucial for synaptic plasticity, a process underlying learning and memory. AMPA receptors, composed of subunits GluA1, GluA2, GluA3, and GluA4, facilitate the influx of sodium (Na+) and calcium (Ca2+) ions upon activation, leading to the depolarization of the postsynaptic neuron [3]. The dynamic regulation of AMPA receptor function and density at synapses contributes to synaptic strengthening or weakening, impacting sensory perception and integration. Consequently, changes in AMPA receptor activity can significantly affect sensory experiences, highlighting their importance in understanding sensory processing [4].

Ketamine, initially developed in the 1960s as a derivative of phencyclidine (PCP), was introduced into clinical practice in the early 1970s as an anesthetic agent. Its rapid onset and short duration of action made it valuable in various medical settings, including surgical procedures and pain management [5]. Ketamine is classified as a dissociative anesthetic, known for inducing a trance-like state while providing analgesia, amnesia, and sedation. More recently, ketamine has gained attention for its off-label use in treating major depressive disorder (MDD) and other mood disorders. Its rapid antidepressant effects, often observed within hours of administration, have made it a significant option for patients resistant to conventional antidepressant therapies [6]. Ketamine primarily exerts its effects through NMDA receptor antagonism. By blocking NMDA receptors, ketamine reduces excitatory neurotransmission and influences the brain's balance between excitatory and inhibitory signals. This action is believed to contribute to its anesthetic and antidepressant properties [7]. However, ketamine's effects extend beyond NMDA receptor antagonism; it has been shown to affect other neurotransmitter systems and signaling pathways, including the enhancement of synaptogenesis and increased release of brain-derived neurotrophic factor (BDNF). Recent research suggests that ketamine may also influence AMPA receptors, further complicating its impact on synaptic function and sensory perception [8].

Given ketamine's multifaceted effects, exploring its impact on AMPA receptors and sensory processing is crucial. While substantial research has focused on ketamine's NMDA receptor antagonism and clinical applications in mood disorders, its influence on AMPA receptor function and sensory processing remains less understood. Investigating how ketamine affects AMPA receptors could provide valuable insights into its broader effects on synaptic plasticity and sensory integration. This understanding could aid in developing targeted therapies for sensory processing disorders and optimizing ketamine's clinical use. Thus, this review aims to bridge the gap between ketamine’s known pharmacological effects and its potential impact on sensory processing. It contributes to a comprehensive understanding of its therapeutic and side-effect profiles.

## Review

Sensory processing and the somatosensory cortex

The somatosensory cortex, located in the brain's parietal lobe, is crucial for processing sensory information from the body. It is divided into two major regions: the primary somatosensory cortex (S1) and the secondary somatosensory cortex (S2). S1 is positioned just behind the central sulcus and is responsible for receiving and processing sensory input from the contralateral side of the body [9]. This region is organized somatotopically, meaning that different body parts are represented in specific areas of the cortex, creating what is known as the sensory homunculus. This organization allows for the precise localization of sensory stimuli and is integral to our ability to perceive touch, temperature, and pain [10]. In contrast, S2 is located inferior to S1 and is involved in processing more complex aspects of sensory input. S2 integrates sensory information with emotional and cognitive contexts, linking to areas such as the hippocampus and amygdala. This integration is essential for higher-order processing, allowing us to interpret sensory experiences in relation to memory and emotional significance. Thus, the somatosensory cortex is vital in integrating sensory inputs, enabling effective perception and response to our environment [11].

AMPA receptors are a type of ionotropic glutamate receptor responsible for mediating fast synaptic transmission in the CNS. Composed of four subunits - GluA1, GluA2, GluA3, and GluA4 - AMPA receptors are characterized by their permeability to sodium (Na+) and potassium (K+) ions. When glutamate binds to these receptors, it triggers the opening of the ion channel, leading to depolarization of the postsynaptic neuron [12]. This rapid communication between neurons is fundamental to various brain functions, including sensory processing, motor control, and cognitive functions. AMPA receptors also play a critical role in synaptic plasticity, particularly in the mechanisms underlying long-term potentiation (LTP), which is essential for learning and memory. Inserting AMPA receptors into the postsynaptic membrane strengthens synaptic connections, enhancing synaptic transmission efficacy. This process is vital for encoding memories and adapting neural circuits in response to experiences. Therefore, AMPA receptors are crucial for fast synaptic transmission and the dynamic changes in synaptic strength that underpin learning and memory formation [13].

The modulation of AMPA receptors has significant implications for synaptic strength and sensory processing. Enhancing AMPA receptor activity can lead to increased synaptic efficacy, which is particularly relevant in sensory perception. For instance, drugs that potentiate AMPA receptor function, such as ketamine, have been shown to enhance sensory processing by increasing the responsiveness of neurons in the somatosensory cortex. This potentiation can improve the detection and discrimination of sensory stimuli, making it a valuable area for therapeutic exploration [4]. Moreover, the influence of AMPA receptor modulation on sensory processing extends to potential treatments for sensory processing disorders and mood disorders associated with altered sensory perception. By understanding how AMPA receptors can be targeted to enhance synaptic transmission and sensory integration, researchers can develop innovative strategies for addressing various neurological and psychiatric conditions. In summary, the somatosensory cortex, with its structured organization and interaction with AMPA receptors, plays a crucial role in integrating and perceiving sensory information, with significant implications for understanding sensory processing and developing therapeutic interventions [14]. Key aspects of sensory processing and the role of the somatosensory cortex are detailed in Table 1.

**Table 1:** Key aspects of sensory processing and the role of the somatosensory cortex AMPA: α-amino-3-hydroxy-5-methyl-4-isoxazolepropionic acid

Aspect	Description
Sensory processing [15]	The method by which the brain receives, interprets, and responds to sensory information from the external and internal environment. This process involves the integration of signals from various sensory modalities.
Somatosensory cortex [16]	A region of the cerebral cortex located in the postcentral gyrus of the parietal lobe, primarily responsible for processing somatosensory information such as touch, pressure, pain, and temperature.
Primary somatosensory cortex (S1) [17]	Divided into four Brodmann areas (3a, 3b, 1, and 2), each processing different types of sensory information. Area 3b is primarily responsible for tactile stimuli, while 3a processes proprioceptive information from muscles and joints. Areas 1 and 2 integrate more complex aspects of sensory input, such as texture and size.
Secondary somatosensory cortex (S2) [11]	Located in the parietal operculum, S2 receives inputs from S1 and is involved in higher-order processing of somatosensory information, such as recognizing the texture and shape of objects. It also plays a role in sensorimotor integration and tactile learning.
Sensory receptors [18]	Specialized receptors (e.g., mechanoreceptors, thermoreceptors, nociceptors) located in the skin, muscles, and other tissues that transduce physical stimuli into electrical signals sent to the brain for interpretation.
Neuroplasticity in sensory processing [19]	The somatosensory cortex exhibits a high degree of neuroplasticity, allowing it to reorganize and adapt to changes in sensory input. This plasticity is crucial for learning new skills, recovering from injuries, and adapting to environmental changes.
Topographic organization [19]	The somatosensory cortex is organized somatotopically, meaning that different cortex regions correspond to specific body parts. This is often depicted by the "homunculus," a distorted representation showing the proportional representation of different body parts based on sensory input density.
Role in sensory discrimination [20]	The somatosensory cortex plays a critical role in distinguishing between different types of stimuli, such as varying textures or pressure levels. This ability is crucial for tasks that require fine motor skills and precision.
AMPA receptors and synaptic transmission [21]	AMPA are glutamate receptors involved in fast excitatory synaptic transmission in the central nervous system. In the somatosensory cortex, AMPA receptor activity is crucial for synaptic plasticity, learning, and memory, and for enhancing sensory processing capabilities.
Clinical relevance [22]	Dysfunction in sensory processing within the somatosensory cortex can lead to sensory disorders, such as tactile defensiveness, chronic pain syndromes, or phantom limb sensations. Understanding the role of the somatosensory cortex in sensory processing is key to developing interventions for these conditions.

Ketamine and AMPA receptor potentiation

Ketamine is well-known as a noncompetitive antagonist of the N-methyl-D-aspartate (NMDA) receptor, which plays a critical role in excitatory neurotransmission. By binding to the NMDA receptor's ion pore, ketamine inhibits the influx of calcium ions, thereby reducing neuronal excitability [23]. This mechanism is central to its anesthetic and analgesic effects. However, ketamine's pharmacological profile extends beyond NMDA receptor antagonism. Notably, it also significantly modulates other glutamate receptors, particularly the AMPA receptors, which are crucial for synaptic plasticity and neuronal communication [24]. The effects of ketamine on glutamatergic signaling are profound. NMDA receptor blockade leads to increased synaptic release of glutamate, which preferentially activates AMPA receptors. This enhanced activation is thought to be a key factor in the rapid antidepressant effects of ketamine and its potential influence on sensory processing. By promoting AMPA receptor activity, ketamine enhances synaptic transmission and increases the overall excitatory tone in the brain [25]. Preclinical studies have demonstrated that ketamine affects AMPA receptor function in significant ways. Research shows that ketamine increases the density of AMPA receptors in critical brain regions, such as the prefrontal cortex and hippocampus, and enhances their functional activity [26]. This potentiation is essential for synaptic efficacy and neuroplasticity, which are important for mood regulation and cognitive function. Ketamine facilitates the insertion of new AMPA receptors into the synaptic membrane and activates downstream signaling pathways, including BDNF and the mammalian target of rapamycin (mTOR). These pathways are crucial for supporting synaptic growth and resilience [27]. The potentiation of AMPA receptors has significant implications for sensory processing. Enhanced AMPA receptor function can improve sensory processing capabilities by increasing excitatory neurotransmission and allowing the brain to process sensory information more effectively. This improvement may result in heightened sensory perception and better sensory input integration, which could benefit individuals with sensory processing disorders [28]. Clinical observations support the idea that ketamine treatment can alter sensory perception. Patients frequently report heightened awareness and altered sensory experiences following ketamine administration. These changes may be linked to the increased synaptic strength and plasticity resulting from AMPA receptor potentiation, which enhances the brain's responsiveness to sensory stimuli. In summary, ketamine's impact on AMPA receptor function not only contributes to its antidepressant effects but also suggests potential therapeutic benefits for sensory integration issues, highlighting its versatility as a treatment option [29].

Clinical evidence and applications

**Figure 1:**
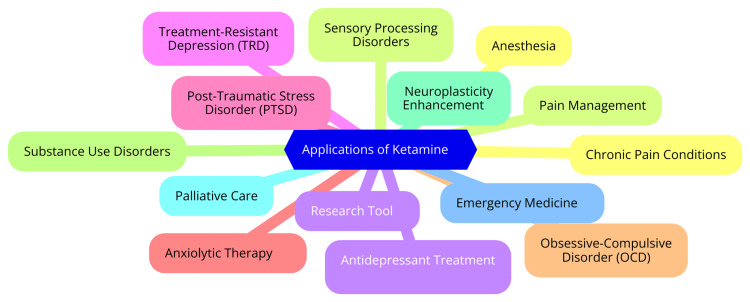
Applications of ketamine Image credit: Dr. Kaustuv Das

Ketamine has garnered significant attention in clinical settings for its potential in treating chronic pain and various sensory-related disorders. Its unique mechanism of action involves antagonizing NMDA receptors while potentiating AMPA receptors, leading to notable changes in sensory processing [30]. Research indicates that low-dose ketamine can offer relief for chronic pain conditions, such as neuropathic pain and fibromyalgia. By modulating the CNS's response to pain stimuli, ketamine alleviates pain and may enhance the overall sensory experience for patients enduring these debilitating conditions. Additionally, ketamine is being investigated for its potential benefits in sensory processing disorders, including complex regional pain syndrome (CRPS), where its effects on synaptic plasticity and sensory gating could help alleviate associated symptoms [31]. Clinical trials have provided valuable insights into ketamine's impact on sensory perception. Patients undergoing ketamine treatment have reported notable changes in their sensory experiences, particularly in tactile and auditory processing [32]. For example, some studies suggest that ketamine administration can alter patients' perception of touch, potentially due to its modulation of sensory processing pathways in the brain. However, findings are inconsistent; some research has shown no significant differences in tactile detection thresholds between self-touch and external touch during ketamine administration [33]. This suggests that while ketamine can influence sensory perception, it may not necessarily affect basic tactile discrimination. Furthermore, the dissociative effects of ketamine can lead to temporary alterations in sensory input perception, presenting intriguing possibilities for addressing complex sensory processing issues in various psychiatric conditions [33]. Compared to traditional treatments for sensory processing disorders, such as cognitive-behavioral therapy (CBT) or pharmacological interventions like selective serotonin reuptake inhibitors (SSRIs), ketamine offers a distinct advantage due to its rapid onset of action. Conventional treatments often require weeks to months to show effects, whereas ketamine can provide immediate relief, making it particularly appealing for acute episodes of sensory distress [34]. However, ketamine's side effects, including dissociation and the potential for abuse, pose challenges that are less common with traditional therapies. While conventional treatments focus on long-term management and gradual symptom reduction, ketamine's role is more geared towards acute intervention and rapid symptom relief, which may complement existing treatment approaches. Overall, ketamine shows promise in treating sensory processing disorders through its analgesic and sensory modulation effects, providing a rapid alternative to traditional therapies. Further research is needed to assess its long-term efficacy and safety in this context [35]. The applications of ketamine are illustrated in Figure 1.

Mechanistic insights

The potentiation of AMPA receptors by ketamine involves complex molecular mechanisms essential for synaptic plasticity and LTP. A critical event in LTP is the recruitment of AMPA receptors to the postsynaptic membrane, which is facilitated by the exocytosis of these receptors from recycling endosomes. This mobilization is triggered by activity-dependent signals, with calcium influx through NMDA receptors activating calcium-sensing proteins, such as synaptotagmin, to promote AMPA receptor exocytosis [36]. The phosphorylation of AMPA receptor subunits, particularly GluA1, enhances their conductance and stability at the synaptic membrane. Protein kinases, such as calcium/calmodulin-dependent protein kinase II (CaMKII), are involved in this phosphorylation process, which is crucial for sustaining LTP. The subunit composition of AMPA receptors also affects their properties and trafficking: GluA2-containing receptors are associated with reduced calcium permeability while GluA1-containing receptors enhance synaptic strength. The dynamic exchange of these subunits at the synapse is vital for modulating synaptic efficacy [37]. AMPA receptors function in concert with other neurotransmitter systems. For instance, glutamate release activates both AMPA receptors and NMDA receptors, making calcium influx critical for LTP induction. Additionally, neuromodulators, such as serotonin, can influence AMPA receptor-mediated transmission, underscoring the complexity of synaptic modulation in response to various neurotransmitters [38]. The potential for synergistic effects exists when ketamine is combined with other pharmacological agents. Agents that enhance NMDA receptor activity might complement the AMPA receptor potentiation induced by ketamine, potentially leading to more robust synaptic strengthening. Similarly, combining ketamine with other antidepressants or anxiolytics could improve therapeutic outcomes by targeting multiple pathways involved in mood regulation. Future research should investigate the efficacy of combination therapies that target both AMPA receptor and NMDA receptor pathways, examining the optimal timing and dosage of these combinations to maximize therapeutic effects for conditions such as depression and anxiety. Mechanistic insights into ketamine's effects on AMPA receptor activity and sensory processing in the somatosensory cortex are detailed in Table 2.

**Table 2:** Mechanistic insights into ketamine's effects on AMPA receptor activity and sensory processing in the somatosensory cortex NDMA: N-methyl-D-aspartate; AMPA: α-amino-3-hydroxy-5-methyl-4-isoxazolepropionic acid; BDNF: Brain-derived neurotrophic factor; mTOR: Mammalian target of rapamycin; MAPK: Mitogen-activated protein kinase

Mechanistic insight	Description
NMDA receptor antagonism [39]	Ketamine primarily acts as an antagonist at NMDA receptors, leading to reduced glutamate activity and altered synaptic signaling. This indirectly influences AMPA receptor function.
AMPA receptor potentiation [25]	Ketamine induces potentiation of AMPA receptors, enhancing their responsiveness and increasing synaptic strength. This effect is thought to be mediated through indirect pathways, including activating signaling cascades.
BDNF upregulation [40]	Ketamine can increase BDNF levels, which in turn promotes AMPA receptor insertion and synaptic plasticity.
Increased synaptic plasticity [41]	Through AMPA receptor potentiation, ketamine enhances synaptic plasticity in the somatosensory cortex, which may lead to improved sensory processing and heightened synaptic responses.
Altered neuronal firing patterns [42]	Ketamine affects the firing patterns of neurons in the somatosensory cortex, potentially leading to changes in how sensory information is processed and perceived.
Molecular changes in AMPA subunits [43]	Ketamine induces changes in the expression or function of AMPA receptor subunits, which can affect receptor kinetics and synaptic transmission.
Signaling pathways [44]	Ketamine influences intracellular signaling pathways (e.g., mTOR, MAPK) that affect AMPA receptor activity and synaptic function.
Effects on glutamate release [45]	By modulating NMDA receptor activity, ketamine indirectly affects glutamate release, which can influence AMPA receptor activity and overall sensory processing.

Future research directions

As the understanding of ketamine's role in enhancing sensory processing through AMPA receptor potentiation evolves, several key areas warrant further investigation. This section outlines existing knowledge gaps, the potential for novel therapeutic approaches, and considerations for the long-term impact and safety of ketamine use [46]. While ketamine's potentiation of AMPA receptors is well-documented, the precise molecular mechanisms behind this effect are not fully understood. Future research should aim to elucidate the roles of specific AMPA receptor subunits, such as GluA1 and GluA2, in ketamine's impact on synaptic plasticity. Additionally, a deeper understanding of the intracellular signaling pathways activated by AMPA receptor potentiation, and their effects on neuronal function, is essential for a comprehensive grasp of ketamine's action [4]. Another significant area of inquiry is the variability in individual responses to ketamine treatment. Genetic factors may influence the variability in efficacy and safety among different individuals. Identifying genetic polymorphisms that affect ketamine's response could lead to more personalized treatment approaches. Furthermore, examining how clinical characteristics, such as age, sex, and comorbidities, influence treatment outcomes is crucial for optimizing therapeutic strategies [4]. While most studies have focused on the hippocampus and prefrontal cortex, the effects of ketamine on other brain regions, such as the somatosensory cortex, remain underexplored. Investigating regional differences in AMPA receptor modulation could provide insights into the broader implications of ketamine treatment and its impact on sensory processing [47]. The mechanisms by which ketamine enhances AMPA receptor activity open up possibilities for developing new treatments. One promising avenue is the development of AMPA receptor modulators that selectively enhance receptor function without the side effects associated with ketamine. Additionally, combining ketamine with other pharmacological agents targeting different neurotransmitter systems may yield synergistic effects, potentially improving treatment outcomes for patients with mood disorders [25]. As research progresses, personalized treatment strategies could be developed by identifying biomarkers that predict which patients are most likely to benefit from ketamine or AMPA receptor-targeting therapies. Depending on individual patient profiles, tailoring dosing regimens may optimize therapeutic outcomes and enhance treatment efficacy [48]. Despite ketamine's demonstrated rapid antidepressant effects, its long-term safety profile requires scrutiny. Future studies should investigate potential neurotoxic effects of prolonged ketamine use, particularly concerning cognitive function and brain structure. Additionally, assessing the long-term psychological impact of ketamine treatment, including risks of dependency or adverse psychiatric effects, is crucial for ensuring patient safety [49]. Understanding the optimal duration and frequency of ketamine administration is also important for maximizing benefits while minimizing risks. Research should explore the efficacy and safety of intermittent versus continuous ketamine treatment for sustaining therapeutic effects. Furthermore, investigating potential withdrawal symptoms or rebound depression following cessation of ketamine therapy will provide valuable insights into the long-term management of patients undergoing treatment [34]. Future research directions regarding ketamine's role in AMPA receptor potentiation and sensory processing are detailed in Table 3.

**Table 3:** Future research directions regarding ketamine's role in AMPA receptor potentiation and sensory processing AMPA: α-amino-3-hydroxy-5-methyl-4-isoxazolepropionic acid; PTSD: Post-traumatic stress disorder

Research direction	Description	Potential impact
Optimizing ketamine dosing [50]	Investigate the optimal dosing regimens for maximizing AMPA receptor potentiation while minimizing adverse effects.	Enhanced therapeutic efficacy with reduced risk of dissociative side effects.
Development of novel AMPA potentiators [46]	Explore new compounds targeting AMPA receptors with similar or improved efficacy to ketamine.	Creation of targeted therapies with potentially fewer side effects or improved outcomes.
Mechanistic studies of AMPA receptor subunits [51]	Examine how ketamine affects different AMPA receptor subunits and their role in sensory processing and neuroplasticity.	Better understanding of specific molecular mechanisms involved in ketamine’s effects.
Long-term effects of ketamine on sensory processing [52]	Assess the long-term impact of ketamine treatment on sensory processing and neuroplasticity, including potential lasting changes in synaptic strength.	Insights into the sustainability of therapeutic effects and potential long-term benefits or risks.
Comparative studies with other glutamatergic agents [53]	Compare ketamine’s effects on AMPA receptors with other glutamatergic agents or modulators to understand relative efficacy and safety profiles.	Identification of the most effective and safest treatment options for sensory processing disorders.
Synergistic effects with adjunct therapies [54]	Investigate the effects of combining ketamine with other therapeutic agents (e.g., antidepressants, analgesics) on AMPA receptor activity and sensory outcomes.	Potential for developing combination therapies with enhanced efficacy for treating complex conditions.
Patient-specific responses and biomarkers [55]	Explore how individual genetic, epigenetic, or biochemical factors influence responses to ketamine treatment and AMPA receptor potentiation.	Personalized treatment strategies based on individual variability and biomarkers.
Applications in neuropsychiatric disorders [56]	Study the effects of ketamine on AMPA receptors in neuropsychiatric disorders where sensory processing is altered, such as depression or PTSD.	Development of targeted treatments for disorders involving disrupted sensory processing.
Impact on neurodevelopment and aging [25]	Investigate how ketamine-induced AMPA receptor potentiation affects sensory processing across developmental stages and aging populations.	Understanding age-related differences in ketamine’s effects and implications for treatment across the lifespan.
Exploring alternative routes of administration [25]	Evaluate the effectiveness and safety of alternative administration routes for ketamine (e.g., nasal, oral) in enhancing AMPA receptor activity.	Potential for more convenient or safer methods of delivering ketamine-based therapies.

## Conclusions

In conclusion, the exploration of ketamine's effects on AMPA receptor potentiation and sensory processing underscores the complexity of its pharmacological profile and its potential therapeutic applications. While ketamine is well-established for its NMDA receptor antagonism and rapid antidepressant effects, its influence on AMPA receptors and sensory integration reveals additional dimensions of its action that warrant further investigation. Understanding how ketamine modulates AMPA receptor function can provide insights into its broader impacts on synaptic plasticity and sensory perception, potentially leading to novel therapeutic approaches for sensory processing disorders and enhancing current treatment strategies. As research continues to uncover the full spectrum of ketamine's effects, it will be crucial to refine its clinical applications and address the challenges associated with its use. This review highlights the need for ongoing studies to elucidate the mechanisms underlying ketamine's impact on sensory processing, aiming to optimize its therapeutic benefits while mitigating adverse effects.
